# Electric Field-Induced Release and Measurement Liquid Biopsy of Urinary Transcriptomics and Key Proteins from Normal and High-risk Pregnant Women

**DOI:** 10.1007/s43032-026-02133-4

**Published:** 2026-06-19

**Authors:** Shubhamoy Ghosh, Shanthie Thamotharan, Giorgia del Vecchio, Carla Janzen, Fang Wei, Sherin U. Devaskar

**Affiliations:** 1 Departments of Pediatrics, David Geffen School of Medicine at UCLA, Los Angeles, CA 90095, USA; 2 Obstetrics & Gynecology, David Geffen School of Medicine at UCLA, Los Angeles, CA 90095, USA; 3 School of Dentistry, UCLA, Los Angeles, CA 90095, USA

**Keywords:** Gestational diabetes mellitus, Pre-eclampsia, Urinary transcriptomics, Non-invasive liquid biopsy monitoring, Electric field release and measurement

## Abstract

Noninvasive early detection of patients at the highest risk for the development of adverse pregnancy outcomes (APOs) such as gestational diabetes mellitus (GDM), pre-eclampsia (PE) and gestational hypertension (gHTN) remains a major challenge. Current screening approaches, including maternal blood tests and ultrasound, are limited by either cost, invasiveness, need for specialized skills, or insufficient predictive accuracy. We tested the hypothesis that a novel liquid biopsy approach using Electric Field-Induced Release and Measurement (EFIRM) platform will detect urinary transcripts/proteins in early gestation differentiating patients for the prediction of subsequently developing APOs. In a small prospective study, urine collected temporally from consented pregnant subjects who later developed GDM (*n* = 12), PE (*n* = 12), or gHTN (*n* = 11), were compared to subjects who never developed APOs (Controls [CON], *n* = 15). Isolated cell-free RNA, subjected to gold standard RNA-sequencing with differential abundances were assessed (both p-adjusted and p-values), and identified early transcriptomic signatures of these APOs. Using EFIRM-derived transcripts we validated these candidate genes and assessed corresponding protein signals. We next developed logistic regression models with leave-one-out cross-validation to preliminarily predict specific APOs. A panel of urinary transcripts (*IL1A, MAPK7, TSNARE1*) predicted GDM (AUC = 0.96; sensitivity = 0.95, specificity = 0.62, and NPV = 0.95), while a separate panel (*NPIPB4, GSDMD, HLA-DPB1*) predicted PE (area under the curve [AUC] = 0.92; sensitivity = 0.91, specificity = 0.62, and negative predictive value [NPV] = 0.90), both being distinct from gHTN. These results support the potential of EFIRM as a noninvasive, real-time, multiplexed urine liquid biopsy platform for early screening and monitoring of APOs, suggesting its future utility in prenatal care at an early gestational age.

## Introduction

Normal pregnancy is a physiological stress that requires numerous adaptations of the maternal body to support growth and development of the placental-fetal unit throughout the three trimesters and culminating in childbirth [1]. Gestational Diabetes and hypertensive disorders of pregnancy (e.g. preeclampsia and gestational hypertension) are examples of adverse pregnancy outcomes (APOs) that increase risk, necessitating close monitoring to prevent morbidity and mortality to both mother and child [2–5]. Gestational diabetes (GDM) is an APO that can lead to a large for gestational age infant who is at high risk for hypoxic-ischemia and birth trauma without early management [6, 7]. GDM is diagnosed by an oral glucose tolerance test (OGTT) after 24 weeks of pregnancy [8]. There are pre-analytic, analytic and post-analytic factors that can sway the results of the OGTT by calling the accuracy of this test into question [9, 10]. Hypertensive disorders of pregnancy may hold devastating perinatal consequences if not recognized and managed in a timely manner [10–12]. Our group focused on GDM, PE and gHTN to determine panels of biomarkers that may predict their later development, as these APOs could benefit from earlier detection than current clinical diagnosis allows. Currently identifying high-risk pregnancies in the first half of gestation depends on assessment of clinical risk factors, biomarkers and ultrasound examinations: an approach that remains relatively imprecise [9, 11, 13, 14]. Recently protein biomarkers (sFlit:PIGF ratio) have been able to discriminate pregnancies that go on to develop preeclampsia (PE) with severe features within two weeks [13], but this marker is used in the latter half of the pregnancy. In early pregnancy there have been some promising studies using cell-free DNA for prediction of preeclampsia [15] as well. Our group and others have assessed cell-free RNAs, microRNAs and proteins in multiple research protocols to construct transcriptomic [16, 17] and micro-transcriptomic [18, 19] panels that may be predictive in early gestation of the subsequent development of APOs.

Recent studies have begun focusing on urinary biomarkers, such as the Congo Red test to detect unfolded proteins in the diagnosis of preeclampsia [20]. Even the detection of pregnancy early in the first trimester is by a urine test that detects HCG in a filter color reaction [21, 22]. This body of information made us question whether urine can serve as a biological fluid upon which liquid biopsy can be undertaken temporally to detect pregnancy associated complications. We hypothesized that by employing urinary transcriptomics, detection of a panel of transcripts will differentiate and predict the occurrence of normal pregnancies from pregnancies that subsequently develop complications. To test this hypothesis, urinary RNA-sequencing followed by Electric Field-Induced Release and Measurement (EFIRM) based liquid biopsy (eLB) [23–27] was performed, with predictive modelling to determine the confidence in prediction of developing clinical features that are characteristic of adverse pregnancy outcomes (APOs).

## Materials and Methods

### Clinical Trial

PARENTs was a single center prospective clinical trial that was approved by the UCLA Institutional Review Board (IRB-15–1388 & IRB-19–1079; approved initially on 10/2/15 & 7/8/2019), during which women following confirmation of their pregnancy, were consented and recruited into the study in their first trimester of pregnancy and were temporally followed until parturition (2017 to 2019) (clinicaltrials.gov: #NCT02786420). Data was collected in-person at three study visits during pregnancy and again at delivery, in addition to telephone interviews, questionnaire surveys and chart abstraction. Of the total subjects recruited in this study, we enrolled and successfully collected urine for the cell-free transcriptomics study from 12 subjects who subsequently developed GDM, 12 developed PE, 11 developed gHTN, and 6 of these pregnancies were associated with intra-uterine growth restricted fetuses, of which 2 pregnancies overlapped with gHTN. To match these adverse pregnancy outcomes (APO) sample sizes, 15 pregnancies without these complications served as controls. Each APO was defined and managed according to the American College of Obstetricians and Gynecologists (ACOG) practice guidelines [28–30].

GDM was defined as any degree of glucose intolerance with an initial recognition during pregnancy. Most women were diagnosed by the two-step Carpenter-Coustan criteria between 24–28 weeks of gestation. This was based on an initial screen of 50-g glucose challenge test (OGTT). Women with glucose values > 135 mg/dL underwent fasting diagnostic 3-h 100-g glucose tolerance test (OGTT).

PE was defined as blood pressure (BP) of 140/90 or higher on two occasions at least four hours apart after 20 weeks of gestation with previously normal blood pressure, and proteinuria of 300 mg/24 h or more [30, 31]. In the absence of proteinuria, PE was defined as new-onset hypertension with new onset of thrombocytopenia, renal insufficiency (serum creatinine greater than 1.1 mg/dL), impaired liver function (elevated liver transaminases to twice the normal concentration), pulmonary edema, cerebral or visual symptoms.

gHTN is hypertension developing after 20 weeks of gestation and not associated with any of the systemic features of PE. Chronic hypertension, on the other hand, was defined as a blood pressure of 140/90 mm Hg or higher that predates pregnancy or develops before 20 weeks of gestation and was excluded from our study.

### Urine Samples

Urine was processed immediately upon collection, by centrifugation at 16,000Xg for 10 min at 4 °C to remove any residual debris and cells, then aliquoted and stored at −80 °C.

### RNA Extraction and Sequencing

Cell-free RNA was extracted from 4 ml of urine using the QIAamp circulating nucleic acid kit (Qiagen; cat. no. 55114). Total RNA was quantified using the Qubit RNA HS Assay kit (Thermofisher Scientific) and RNA integrity verified using the Agilent High sensitivity RNA Screen Tape System (Agilent) on a 2200 TapeStation System (Agilent). Ten μl of the extracted RNA (ranging from 2–4 ng of total RNA) was used to construct RNA-seq libraries using the ultra-low input specific kit, NuGen Ovation SoLo RNA-Seq kit with Human rRNA AnyDeplete kit (NuGen) according to the manufacturer’s instructions. Libraries were sequenced using 100 bp single-end reads on the Hiseq 4000 System (Illumina).

### RNA Seq Analysis

**Fig. 1:**
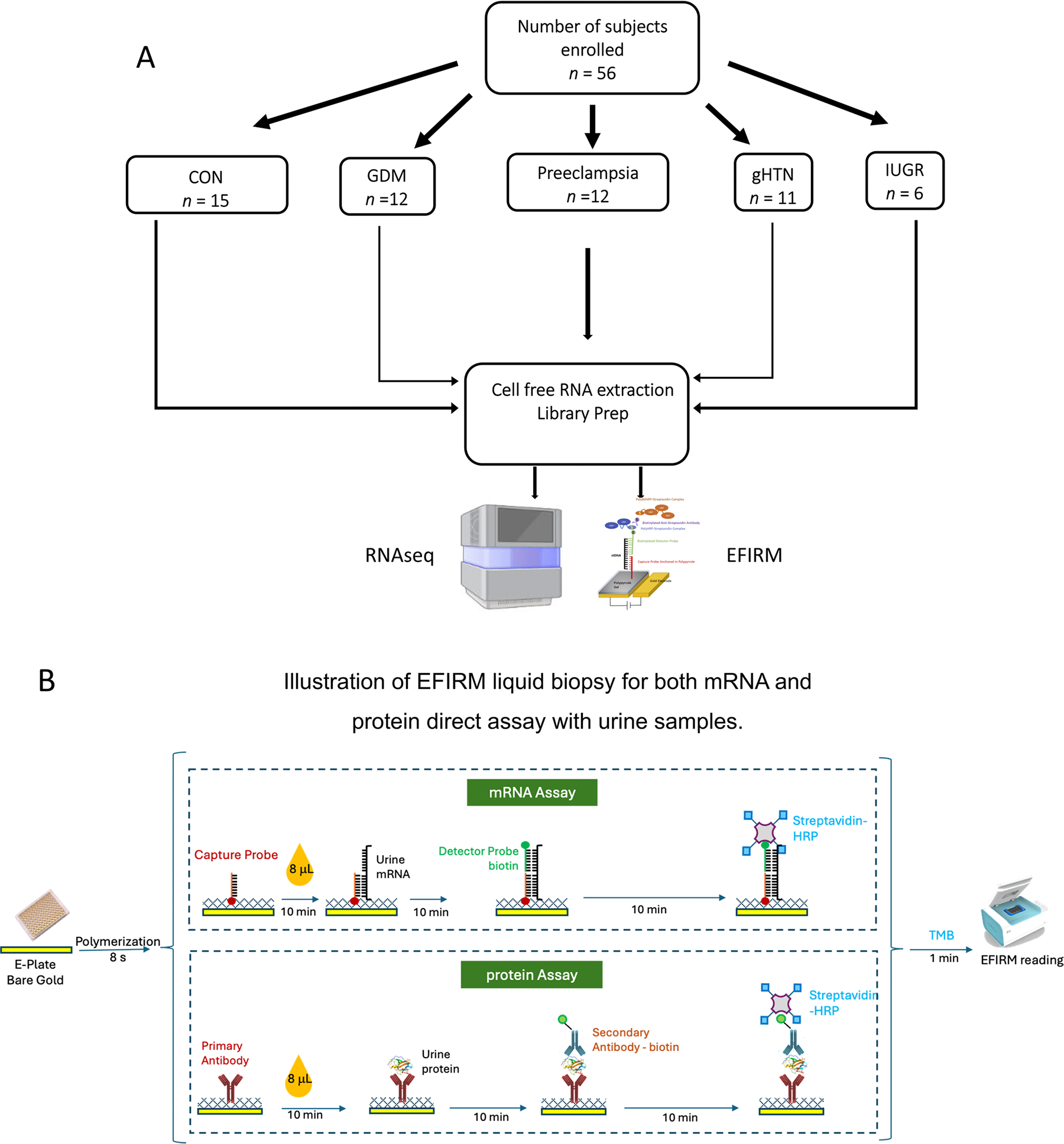
**A**. Schematic representation: illustrating the distribution of biological samples, and the overall experimental design used to study different pregnancy complications. **B.** Illustration of the EFIRM liquid biopsy technology employed for quantification of both mRNA (top panel) and protein (bottom panel) direct assay using urine samples

Raw sequencing reads were initially assessed for quality using FastQC (version v0.118). All samples passed this quality control step before proceeding further with analysis. The demultiplexed fastq files along with other processed files are available at GSE286453. The RNA-seq reads were then aligned to the human reference genome (hg19) using STAR aligner (version v2.5.2b), with only unique alignments and those with up to 4 mismatches being retained. The GeneCounts option in STAR was used to count the number of reads mapped to each gene. For downstream analysis, we used the DESeq2 package (version v1.20.0) in R to normalize the data (via the rlog function) and perform differential gene expression (DEG) analysis. A significance threshold was set with an adjusted *p*-value (FDR) < 0.05 and log2Foldchange ≥ 1. Subsequent Gene Set Enrichment analysis was performed to uncover important biological insights and improve our understanding of diseases. To this end a ranked list of genes was created by multiplying the fold change value by −log10 p-value [32]. Gene Set Enrichment Analysis was performed against MSigDB using GSEA tools from the BROAD Institute (Fig. 1A).

### RT-PCR Validation of DE Genes

RT-PCR validation was undertaken on the extracted RNA which was converted into cDNA and pre-amplified using the Ovation picoSL WTA system V2 (NuGen). We then performed the qRT-PCR with the TaqMan Fast Advanced Master Mix (Applied Biosystems) and the commercially available probes for a few selected genes: IL1A, MAPK7, QSOX1, and TERF2. All the tested gene probes contained the FAM^™^ reporter dye. GAPDH was used as internal control and these probes had the VIC^™^ reporter dye. Thermocycling conditions employed were according to the manufacturer’s standard protocol (Applied Biosystems). Briefly, reactions were subjected to initial denaturation at 95 °C for 20 s (for uracil-DNA-glycosylase activation), followed by 40 cycles of denaturation at 95 °C for 1 s, with annealing and extension at 60 °C for 20 s.

### Classification Models and Early Biomarker Discovery

We used the Log normalized counts obtained with DESeq2 [17] from the cfRNA seq data in the 1 st trimester of pregnancy. Differentially abundant genes were identified by comparing GDM and PE samples to pregnant controls, using a threshold of adjusted P-value ≤ 0.05 and absolute log2 fold change ≥ 1. Feature selection and biomarker discovery were performed using the glmnet package v2.0–16 for R v3.4.4 [33]. Features were selected using logistic regression (LR) with the elastic-net regularization. The model was validated using a leave-one-out cross-validation (LOOCV) and an additional internal sevenfold cross-validation was used to select the penalty parameter, lambda, to minimize the mean absolute error or misclassification error of the predictions. Features with coefficients greater than zero after the elastic-net regularization were selected as potential biomarkers. Receiver operator characteristic (ROC) curves were constructed using the ROCplot package in R [34].

### EFIRM Liquid Biopsy

The EFIRM assay has been previously described [23, 24, 26]. In each EFIRM assay 8 μL of the urine sample was directly mixed with a buffer in a 1:2 dilution with 1 part urine and 2 parts of buffer. Thus 5 μL of 50% GIT (guanidine thiocyanate; Sigma-Aldrich, USA) + 3 M KCl (potassium chloride; Sigma-Aldrich, USA) and 15 μL of CPBS (Blocker casein in PBS, Fisher Scientific) (1:3) made a total of 20 μL of the buffer (1:2).

### EFIRM Transcript Probes

Paired probes (non-labeled capture and biotinylated detector [at the 3’-end] probes; Integrated DNA Technologies, San Diego, CA) specific for the targeted mRNA biomarkers were designed for EFIRM as shown in Table 1. We examined urine samples temporally (trimester [T] 1, 2, 3 and at delivery = 4) from pregnant controls, GDM, PE and gHTN subjects. Based on our initial RNA-seq results with RT-qPCR validation, we initially chose 4 target transcripts for testing in eLB assays. The chosen transcripts were IL1A, MAPK7, QSOX1, TERF2. This initial set was followed by the addition of GSDMD and HLADPB1 which were detected to be differentially expressed by RNA-seq analysis and were part of the biomarker discovery gene panel. In all cases GAPDH served as the internal reference RNA (Table 1).

### mRNA EFIRM Assay

The capture probes (100 nmol/L) were initially co-polymerized with pyrrole onto the bare gold electrodes by applying a cyclic square wave electric field at 350 mV for 1 s and 1100 mV for 1 s for four cycles. The sensor was then washed with 1XPBST buffer (Thermo Fisher, Waltham, MA). Hybridization was performed in 25 μL of pretreated urine samples in a 1:3 dilution by adding 1 part of 50% GIT + 3 M KCl and 3 parts CPBS [27, 35]. The EFIRM conditions consisted of −200 mV for 1 s and 200 mV for 1 s over a total of 150 cycles in each 2 s epoch followed by a 10-min incubation period at room temperature. After washing, the detector probes were mixed with casein-phosphate buffered saline (Invitrogen, Carlsbad, CA) at a 1:100 dilution and then transferred onto the electrodes. Subsequently, streptavidin poly-HRP80 conjugate (Fitzgerald Industries, Acton, MA) was mixed with casein-phosphate buffered saline (Invitrogen) at a 1:3 ratio and incubated for 15 min. Amperometric current was ultimately measured in TMB (3,3’,5′5’-tetramethylbenzidine) at −200 mV for 1 min. The amperometric current reading from the EFIRM is being presented in negative nanoAmpere (nA), and only the nA readings at a steady state noted after 60 s were recorded (Fig. 1B).

### Protein Assay via EFIRM

The EFIRM platform was employed to detect and quantify four target proteins within urine samples. The primary and secondary antibodies, and the protein standards for each target protein were sourced as follows: 1) Anti-GSDMD primary antibody (Catalog #LS-C375837) and the biotin-conjugated secondary antibody (Catalog #LS-C375842; LifeSpan BioSciences; LSBio), and the recombinant protein standard (Catalog #RPC27181; Biomatik). 2) Anti-HLA-DPB1 primary antibody (Catalog #BS-4107R; Bioss via ThermoFisher Scientific), biotin-conjugated secondary antibody (Catalog #LS-C370797; LifeSpan BioSciences; LSBio), and the recombinant protein standard (Catalog #RPC21499; Biomatik). 3) Anti-IL1A primary antibody (Catalog #TA590791; OriGene), biotin-conjugated secondary antibody (Catalog #BAF200; Biotechne/R&D Systems), and the corresponding recombinant protein standard (Catalog #TP72001; OriGene). 4) Anti-MAPK7 primary antibody (Catalog #MAPK7–101AP; ThermoFisher Scientific), the biotin-conjugated secondary antibody (Catalog #TA502203AM; OriGene), and the recombinant protein standard (Catalog #RP50463; OriGene).

The assay utilized a 96-array E-Plate (EzLife Bio. Inc., USA) integrated with gold electrodes. The primary antibody for each protein target was diluted (0.5 μg/mL) and co-polymerized with pyrrole (Sigma-Aldrich, St. Louis, MO) onto the electrode surfaces. This electro-polymerization was performed by applying a cyclic square wave electric field, alternating between 350 mV for 1 s and 1100 mV for 1 s, over four cycles. Following polymerization, six wash cycles with 1 × PBS containing 0.05% Tween-20 (PBS-T) were conducted using a Biotek 405LS plate washer to remove unbound materials. 8 μL of the urine sample that was directly mixed with a buffer in a 1:2 dilution, with 1 part urine and 2 parts of buffer, within 25 μL of the diluted sample was added to each well, followed by a 10-min incubation at room temperature. Unbound components were removed through six additional PBS-T wash cycles. To control the quality of the assay, a serial dilution of each protein with recombinant standards were prepared ranging from 1 μg/mL to 1 pg/mL. After washing off the unbound urine sample with PBS-T, biotin-conjugated secondary antibodies were diluted 1:500 in Casein/PBS, and 30 μL was added to each well, followed by another 10-min of incubation, and then washed. Signal amplification was achieved by incubating the wells with Poly-HRP80 (Fitzgerald Industries, Acton, MA), diluted 1:4 in Casein/PBS, for 10 min. After a final series of wash cycles, 30 μL of 1-Step Ultra TMB (ThermoFisher, Waltham, MA) was added to each well to initiate the electrochemical reaction. The amperometric signal was measured using a 96-array reader (EzLife Bio. Inc., USA) at −200 mV for 60 s, with each well reading taken three times to ensure accuracy (Fig. 1B).

### Statistical Analysis

Statistical analysis was performed using SPSS 23.0 (SPSS Inc.) and the R program. For multi-group or time-based comparisons, ANOVA models followed by Tukey’s test were employed. The Mann–Whitney U test was used for non-paired comparisons of two groups (e.g., GDM versus control and PE/gHTN versus control, or Overweight versus Lean and Obese versus Lean), along with the unpaired Student’s t-test only in the case of quantitative real-time PCR results. Pearson correlation coefficients were obtained for determination of linear relationships between RNA-seq and EFIRM platforms. All the p values were set for a threshold of significance (α) at 0.05 unless specified otherwise. The adjusted P values in RNA sequencing analysis were calculated in DESeq2 [17] that used Benjamin-Hochberg correction [36].

## Results

### Subject Characteristics

The consented subjects who were recruited for this single center study analyses (total *n* = 56) are depicted in Table 2, along with the clinical features within the three groups who presented with APOs (*n* = 41), separated as GDM (*n* = 12), PE (*n* = 12), and gHTN (n-11). There were 6 offspring with IUGR (*n* = 6) with two subjects overlapping with those who developed gHTN, along with unaffected pregnancies (*n* = 15) serving as the gold standard control. As is evident, the subjects who developed GDM had higher glucose values, while both PE and gHTN revealed higher systolic and diastolic blood pressures, when compared to the normal group (Table 2). Both pre-pregnancy body mass indices (BMI), hemoglobin A1C (HbA1C), and first to early second trimester blood pressures (BP) were normal and no difference between the groups emanated (Table 2), supporting the development of glucose abnormalities in the GDM and aberrant BP in PE and gHTN only in late second or third trimesters of pregnancy, attesting that these are not pre-existing but rather gestational conditions. While no statistical differences in gestational age at delivery, all being at term, and the associated birth weights were seen, trend towards a decline is seen in gHTN more so than in PE. Similar trends are seen with urinary protein concentrations and the protein/creatinine ratios, increasing only in the PE group, with no changes observed with the liver function tests and circulating platelet counts in any of the groups (Table 2).

### Urine-DERIVED mRNA Profiles Associated with Differing Adverse Pregnancy Outcomes

**Fig. 2:**
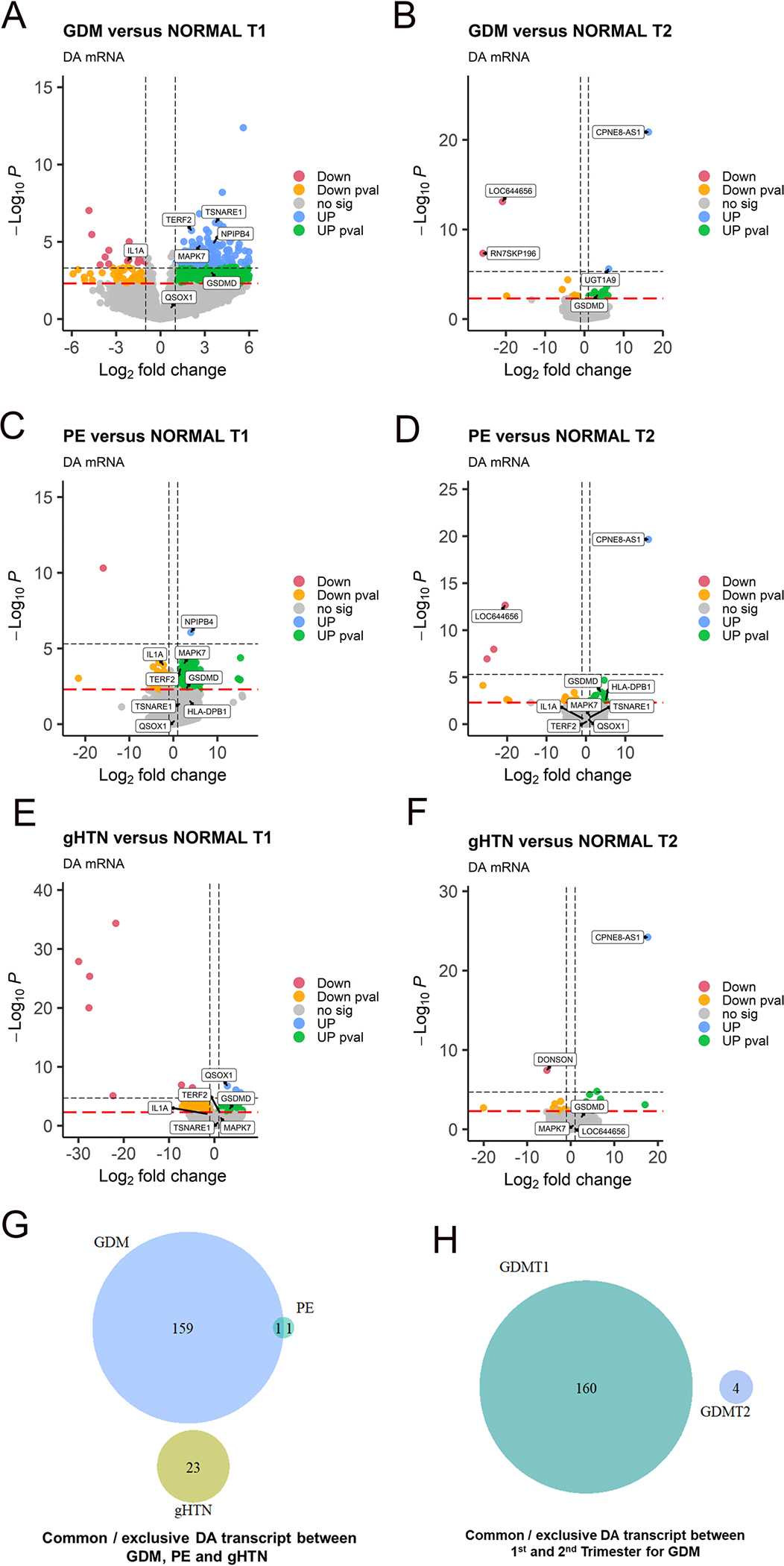
Differentially Abundant Transcripts: Volcano plots depicting differentially abundant transcripts isolated from urine samples collected from subjects who subsequently developed gestational diabetes mellitus (GDM) (**A-B**), preeclampsia (PE) (**C-D**), and gestational hypertension (gHTN) (**E–F**) during the 1 st (**A, C, E**) and 2nd (**B, D, F**) trimesters, when compared to control (CON) pregnancies. The fold change for each transcript/gene is plotted on the x-axis, while statistical significance is plotted on the y-axis. Transcripts/genes that were upregulated with a fold change ≥ 2 and a padj < 0.05 are shown in blue, and those that were downregulated are shown in red. Transcripts/genes upregulated with a fold change ≥ 2 and a *p*-value < 0.005 are highlighted in green, while those downregulated with the same fold change and *p*-value < 0.005 are shown in yellow. Gray represents genes that did not show significant differences based on the previously mentioned criteria. In panels (**G-H**), Venn diagrams are presented to demonstrate the overlap of transcripts across various pregnancy complications during the 1 st trimester (**G**), as well as exclusive transcripts between the 1 st and 2nd trimesters of GDM compared to CON (**H**)

Employing a non-invasive approach of urine derived mRNA sequencing, we identified differentially abundant (DA) transcripts with a primary cutoff fold change (-FC) of > 2 and a *p*-adjusted value of < 0.05. Given the sample size/group, we also applied a secondary cutoff of using the raw *p*-value of < 0.005 to explore trends in transcript abundance of biological relevance across the different APOs. When comparing GDM to control pregnancies, during T1, 160 DA transcripts were identified (*p*-adjusted value = < 0.05), and 593 (*p*-value of < 0.005) (Fig. 2A); while in T2, we identified 4 and 36, respectively (Fig. 2B). The corresponding heat maps demonstrating DA genes between GDM and control pregnancies during T1 and T2 are shown in Supplemental Fig. 1A. In the case of PE, 2 and 4 DA transcripts were observed (*p*-adjusted value of < 0.05), while 341 and 57 were detected respectively (at a *p*-value of < 0.005) (Fig. 2C, D). The corresponding heat maps are shown in Supplemental Fig. 1B. In the case of gHTN, 23 and 2 DA transcripts were identified (*p*-adjusted value of < 0.05), while 337 and 45 were respectively observed (at a *p*-value < 0.005) during T1 and T2 (Fig. 2E, F). The corresponding heat maps are seen as a Supplemental Fig. 1C. The total numbers of genes that are different between GDM, PE and gHTN (all APOs) versus control pregnancies during T1 alone are depicted as Venn diagrams (Fig. 2G) with a similar depiction of the differences between GDM alone versus control pregnancies during T1 and T2 (Fig. 2H).

### Molecular Pathways

**Fig. 3:**
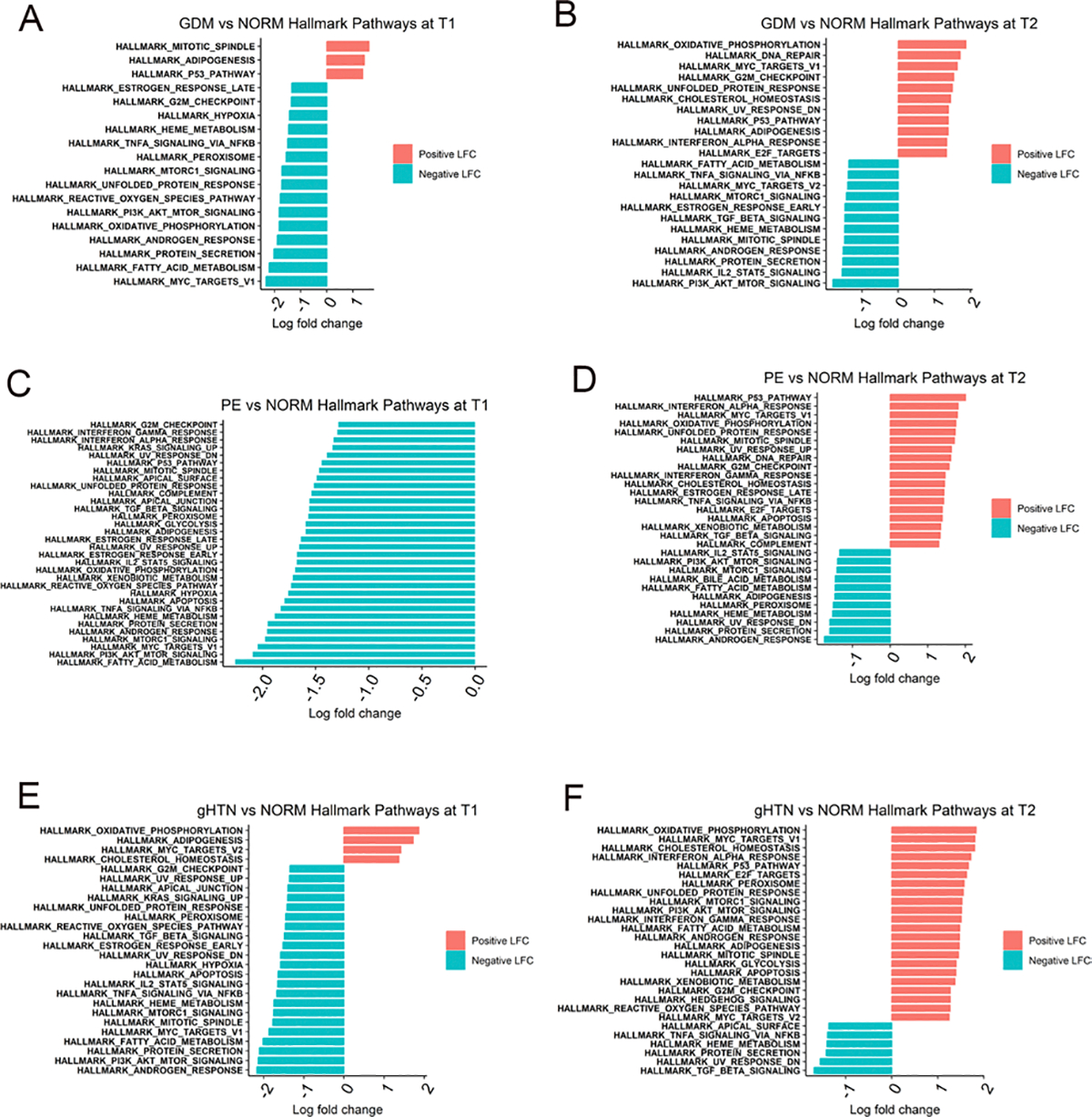
Pathway analysis: bidirectional box plots showing list of hallmark pathways that were positively (red) or negatively (blue) enriched between GDM vs CON (**A, B**), PE vs CON (**C, D**) and gHTN vs CON (**E, F**) during first (**A, C, E**) and second (**B, D, F**) trimesters. X-axis shows the log-fold change, and the hallmark pathways are plotted on the y-axis

Next, we undertook molecular pathway analysis (Fig. 3) for GDM versus control pregnancies, during T1 (Fig. 3A) and T2 (Fig. 3B). During both trimesters, pathways of adipogenesis and p53 positively enriched, while mTOR and fatty acid metabolism negatively enriched in GDM versus control pregnancies in both trimesters (T1 and T2). Similar changes were evident in PE during T1 and T2. During T1 most pathways identified were diminished compared to control pregnancies and overlapped with the reduction seen in T2 as well, involving fatty acid metabolism and mTOR pathways (Fig. 3C, D). A positive enrichment in pathways was only seen during T2 that involved P53, unfolded protein response, cholesterol metabolism and interferon gamma response pathways (Fig. 3D), as some of these pathways were negatively enriched during T1 (Fig. 3C). Further, cell cycle entry point G2/M and DNA repair pathways were enriched with associated mitotic pathway and cellular apoptotic pathway changes most prominently featured during T2, only to be negatively enriched during T1. On the other hand, when assessing DA molecular pathways in gHTN compared to control pregnancies (Fig. 3E, F), during T1 (Fig. 3E) a reduction in mTOR and fatty acid metabolism was again evident along with a reduction in the TNFα pathway. In contrast an enrichment in oxidative phosphorylation, adipogenesis, and certain inflammatory pathways emerged. Of particular interest when comparing PE and gHTN to control pregnancies, pathways reduced during T1 were observed to escalate during T2, suggestive of an adaptive phenomenon being displayed with APOs.

### Differentially Abundant Genes

**Fig. 4:**
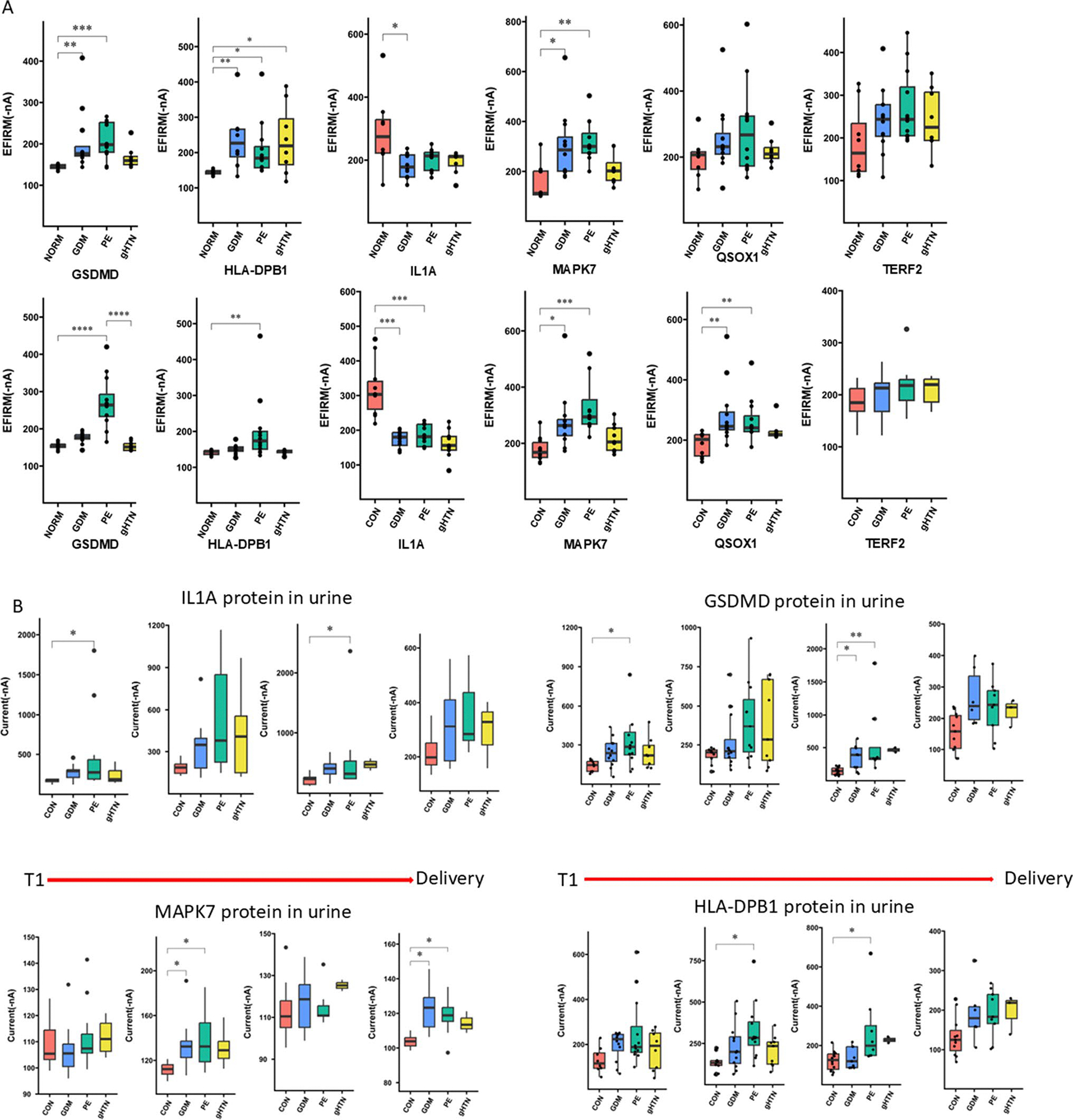
Electric field–induced release and measurement liquid biopsy (eLB): Validation of differentially abundant transcripts (**A**) and proteins (**B**) from urine samples. Corresponding probes to detect transcripts were applied to the electrochemical sensor to detect GSDMD, HLA-DPB1, IL1A, MAPK7, QSOX1 and TERF2 at trimester 1 (top panel) and trimester 2 (bottom panel) across all pregnancy complications compared to control pregnancies (**A**). Corresponding antibodies were applied to the electrochemical sensor to detect IL1A, GSDMD (top panels), MAPK7, HLA-DPB1 (bottom panels) during trimesters 1,2,3 and during delivery (graphs from left to right) (**B**). *P* < *0.05, ** < 0.01, *** < 0.001 and **** < 0.0001

Following delineation of molecular pathways that were perturbed in GDM, PE and gHTN during T1 and T2, we next focused on specific genes that were differentially abundant in the different APOs during T1 and T2, thereby facilitating the required validation by other methods such as RT-qPCR which demonstrated parallel changes or trends (Supplemental Fig. 2), and a relatively novel EFIRM liquid biopsy technology (Fig. 4). We observed changes consistently with interleukin 1 A (IL1A; RNAseq: 2.2-fold; *p*-adjusted value = 0.04) and the mitogen-activated protein kinase 7 also referred to as ERK5 (MAPK7, RNAseq: 2.7-fold, *p*-adjusted value = 0.01) (Fig. 4A). During both T1 and T2, a reduction in IL1A (inflammation) transcripts emerged in GDM and PE versus control pregnancies. In contrast, an increase was seen with MAPK7 (signaling molecule mediating cellular proliferation and determining cell-fate) in both GDM and PE during T1 and T2 (Fig. 4A). Such changes were absent in gHTN during both T1 and T2. Given that the reduction in urine IL1A was against the expected inflammatory state associated with APOs, we further confirmed this observation by measuring the corresponding proteins by EFIRM. In distinct contrast to the reduced IL1A transcript, an increase in the IL1A protein was observed in PE during all trimesters, achieving statistical significance during T1, T3, T4, with only a trend seen during T2 (Fig. 4B). No such protein change was evident in the case of GDM or gHTN (Fig. 4B). This discrepancy between transcript and protein level of IL1A can be a result of either enhanced translation or it could reflect diminished RNA stability or enhanced secretion of the protein in the urine. (Fig. 4 A, B). In the case of MAPK7 protein concentrations, however, they increased during T2 and T4 in both GDM and PE, the mRNA content paralleling the protein concentrations (Fig. 4 A, B). In addition to these two transcripts with their translated products, two additional transcripts were examined. These two transcripts were selected based on their DA but also on prior reports demonstrating their involvement in GDM [37, 38]. This selection was deliberate to confirm the detectability of change by EFIRM of transcripts in biofluids. To this end, Gasdermin D (GSDMD) that gives rise to a pore-forming protein playing a key role in systemic defense against infection and danger signals as part of inflammasomes required for pyroptosis [37, 38], and the human leukocyte antigen HLA-DPB1 that participates in the formation of the antigen-binding DPab heterodimer that triggers systemic immune response [39], when assessed were observed to increase in GDM and PE particularly during T1, while only increasing in PE during T2 (Fig. 4A). Again, no change in these two transcripts was observed with gHTN during T1 and T2, except HLA-DPB1 that increased only during T1. Additionally, the Quiescin sulfhydryl oxidase 1 (QSOX1) that is located within Golgi and present in secreted biofluids [40, 41], increased during T2 in both GDM and PE. Finally, telomeric repeat binding factor 2 (TERF2) which is a component of the telomere nucleoprotein complex [42, 43], portrayed a higher trend only during T1, in GDM, PE and gHTN, with a lack of change during T2 (Fig. 4A).

Two additional transcripts, the nuclear pore complex interacting protein (NPIPB4) family member [44] in the case of PE (RNAseq: 3.6-fold change; *p* adjusted value = 0.01) and TSNARE1 (RNAseq: 3.7-fold change; *p* adjusted vale = 0.002) that codes for an endosomal protein regulating trafficking in various cells including those in placenta and neurons [45], were differentially abundant in the case of GDM (data not shown). Similar analyses in pregnancies carrying FGR/IUGR fetuses were also performed but given the further limitation of sample size, we have not presented these results. Despite major differences between the two methods engaged in assessing urine transcripts, namely RNA-seq and EFIRM, with the former reliant on the efficiency of RNA extraction, library construction and sequencing, while the latter carried out measurements directly in the biofluid, we undertook correlation analyses. The correlation analyses between RNA sequencing and EFIRM, despite the differences, revealed moderate (R = 0.46 to 0.51 for GDM) to moderately strong (R = 0.51 to 0.73 for PE) but significant associations for key transcripts, MAPK7, GSDMD, and IL1A, chosen for their biological relevance in APOs such as GDM and PE (Supplementary Fig. 3).

### Prediction Modelling

**Fig. 5:**
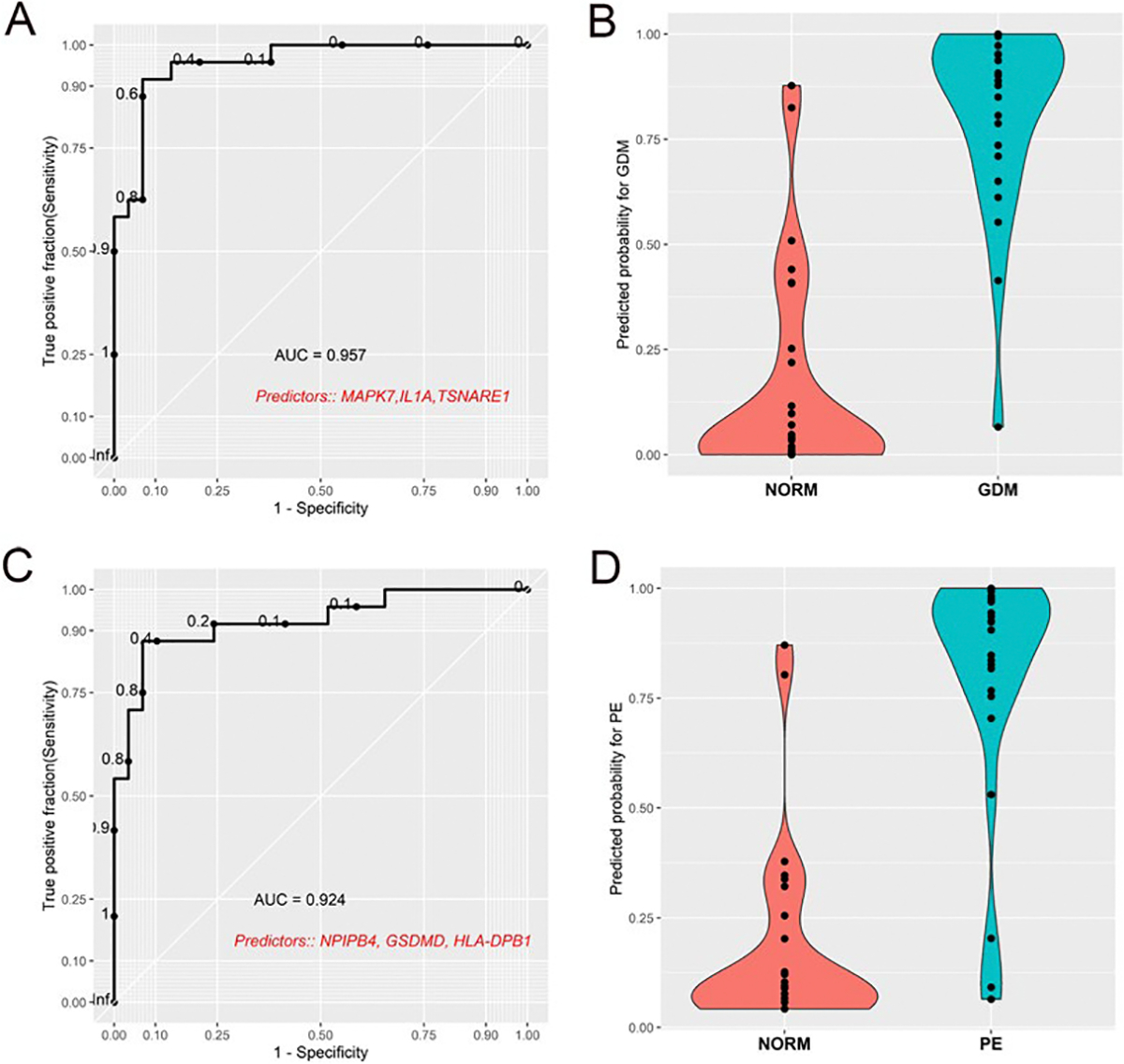
Logistic regression model for prediction of transcripts detected in urine samples: collected from pregnant subjects during the 1 st trimester. Receiver operator characteristic (ROC) curves were generated employing urine mRNAs from GDM (*n* = 12) (**A**) and PE (*n* = 12) (**C**) subjects versus control pregnancies (*n* = 15). Violin plots of predicted probability of mRNAs obtained from GDM (**B**) and PE (**D**) subjects, with 0 = low probability and 1 = high probability

Finally, despite a small sample size in each sub-group, we launched a pilot prediction modelling as a proof-of-principle. Employing Logistic regression with leave-one-out coefficient of variation, prediction of GDM as early as in the 1 st trimester of pregnancy was possible based on a panel of urinary transcripts (IL1A, MAPK7, TSNARE1) resulting in an area under the curve (AUC) for the receiver operator curve (ROC) of 0.96 (Fig. 5A), supporting differentiation between GDM and control pregnancies as seen in the corresponding violin plot (Fig. 5B). Similarly employing a separate urinary panel of transcripts (NPIPB4, GSDMD and HLA-DPB1) that were differentially abundant during T1 in predicting PE displayed an AUC of 0.92 (Fig. 5C), differentiating PE from control pregnancies as seen in the corresponding violin plot (Fig. 5D). Based on the sensitivity (GDM = 0.95, PE = 0.91) and specificity (GDM = 0.62, PE = 0.62) of prediction, we calculated the positive predictive (PPV: GDM = 0.68, PE = 0.66) and negative predictive (NPV: GDM = 0.95, PE = 0.90) values for both GDM and PE (Table 3).

## Discussion

We have for the first time demonstrated the ability to detect a small panel of transcripts and pursuance of the corresponding translated protein products by a rapid testing modality of EFIRM employing urine samples from the first and second trimesters of pregnancy. Examination of proteins demonstrated some variability in alignment with the corresponding transcripts. This was particularly evident with IL1A, an “alarmin” protein released upon cell injury and/or inflammation in the absence of a similar change in the corresponding transcript [46, 47]. This was despite the use of a singular highly sensitive EFIRM detection platform designed to overcome any false discordance due to methodological differences between mRNA and protein, related to detection sensitivity. However, variances in antibody affinities and large sizes of proteins (versus transcripts) with dependency on the efficiency of glomerular filtration and tubular secretion/re-absorption, influence both the presence and concentration of proteins in urine [48]. More often a true discordance between transcripts and corresponding proteins occurs due to biological differences between transcription rates from DNA templates aligning with post-transcriptional processes such as mRNA stability, translation efficiency and protein degradation (as seen with IL1A) [49]. In fact, an increase in IL1A protein seen in APOs may have depleted and reduced the corresponding IL1A mRNA pool. Urinary transcripts and proteins are complementary rather than redundant biomarkers demonstrating the utility of EFIRM in simultaneous multi-analyte detection.

As a proof-of-concept, we also demonstrated the predict-ability of this differentially abundant panel of transcripts with a robust AUC along with sensitivity and specificity, thereby projecting the positive predictive and negative predictive values.

Other studies also employing small sample sizes have detected different metabolites in urine samples that overlap between PE and GDM [50, 51], further there are much larger studies ongoing where urine samples are being collected temporally for future analyses of metabolites [52]. The ImPRoVE study determined difficulty in assessing proteins but were successful in measuring metabolites and certain amino acids in urine samples by the time-consuming LC–MS separation and identification methodology [53]. Given these studies, we focused initially on the transcripts, which were easily detected and measurable in urine. We noted the ability to detect differential abundances between the different APOs, namely GDM, PE, and gHTN.

While RNA-seq served as the gold standard for detection of differentially abundant (DA) transcripts, our attempt at validation by the conventional RT-qPCR led to some variability without statistical significance in most transcripts. However, trending changes emerged in parallel to the changes seen by RNA-seq, except for IL1A which was significantly reduced by RT-qPCR as well, again definitively confirming the observation made by RNA-seq analysis. We next resorted to EFIRM, a relatively novel rapid test that relies on electrochemical fields and noted that the variability was less compared to RT-qPCR quantification. Our study suggests that EFIRM holds promise as a sensitive and versatile approach for urine-based point-of-care (POC) biosensing, particularly beyond conventional pregnancy testing. Traditional POC devices, such as lateral flow assays and electrochemical immunosensors, primarily focus on detecting human chorionic gonadotropin (hCG) concentrations to confirm pregnancy status [54–57]. While these devices are effective for confirming pregnancy, they are generally not optimized for more complex molecular analyses related to pregnancy health and complications [58]. In contrast, EFIRM enables the direct, multiplexed detection of both mRNA transcripts and proteins from unprocessed urine samples. This capability is particularly advantageous for early gestational monitoring, where biomarker concentrations are typically low. Our findings indicate that EFIRM can sensitively and reproducibly detect key transcripts such as IL1A, MAPK7, GSDMD, and HLA-DPB1, which are associated with GDM and PE, as early as in T1. This level of molecular detail is unattainable with conventional POC devices, which are limited to protein detection and often require larger sample volumes and more complex sample pre-preparation [55, 59]. Moreover, EFIRM’s rapid turnaround time and minimal sample requirements make it well-suited for the future integration into clinical workflows, facilitating timely risk stratification and personalized prenatal care.

In building prediction models, it is important to have an intervention that either halts or reverses the disease process from progressing. To date, more so in PE than GDM, there is no known disease reversing intervention, except for the early introduction of aspirin in suspected clinically high-risk patients for developing PE [14]. However, the lack of consistent and reliable technology to detect a panel of biomarkers interferes with stratification of patients when trialing interventions for their success in sub-populations towards detection of endophenotypes. Currently such screening panels of biomarkers much earlier in gestation do not exist beyond the use of sFlt:PIGF ratios known to be useful only two weeks prior to the emergence of PE symptomatology [60], and more recently proven useful in separating PE from PE with severe features [61]. Further, close vigilance with necessary monitoring along with the ability to intervene when indicated can be pursued once a panel of transcripts predict with confidence the subsequent emergence of an APO. By providing a more comprehensive molecular profile from a simple urine sample, EFIRM has the potential to address the unmet need for early, non-invasive, and informative diagnostics, thereby serving as a transformative tool in early risk stratification.

The ability of EFIRM to detect transcriptomic alterations in T1 and T2, well before clinical symptoms of GDM or PE emerge, highlights its potential in altering the standard of care for monitoring pregnancy-related complications. Traditional screening methods, such as OGTT for GDM or sFlt-1/PlGF ratios for PE, are typically implemented in the late second or third trimester, by which time physiological changes may already have become irreversible. Our data shows that transcript panels identified via RNA-seq and validated using EFIRM could distinguish between control and complicated pregnancies as early as the first trimester, with strong negative predictive values. Since these two methods rely on either requiring processing or no processing of the biofluid samples, respectively, a strong correlation between the two is not expected. This is because EFIRM is reliant on direct assessment with no processing, while RNA-seq is accurate but limited by losses during sample preparation, namely RNA extraction, amplification, library construction and sequencing. Thus, EFIRM opens the door for individualized surveillance plans, where patients at high risk could be triaged earlier for lifestyle interventions or pharmacological prophylaxis. Beyond diagnosis, EFIRM’s low sample requirement and non-invasive quality allow for repeated, temporal sampling—making it uniquely suited for dynamic monitoring of pregnancy progression. This may provide clinicians with a window into molecular changes across gestation and enable real-time treatment adjustments. Furthermore, the platform’s portability and scalability lend themselves to global implementation, especially in some sub-populations where access to health care with timely diagnosis and interventions remain challenging, and maternal morbidity and mortality from pregnancy complications remain high. These findings could potentially support the broader vision of EFIRM not only as a diagnostic test but also as a clinical decision-making tool to personalize maternal–fetal care. Prior to realizing such possibilities, future validation in larger multicenter cohorts will be necessary to explore biomarkers of relevance not only to the currently examined but other APOs as well (namely fetal growth restriction and premature birth).

The strength of our present study is that this was a prospectively conducted temporally performed clinical trial, where multiple urine samples at the different trimesters of pregnancy were obtained and analyzed. We were able to detect minimal amounts of transcripts successfully in urine when they are least present in the first trimester, allowing for distinction between APOs and control pregnancies. This ability to bring multiplexing capabilities in rapid detection of a panel of transcripts non-invasively in a biofluid with high sensitivity is a major strength. The additional ability of rapidly screening and predicting with confidence against false negatives, even prior to the emergence of characteristic clinical features of a particular APO, makes this technology ideally suited for clinical settings where decision making occurs in real time.

A limitation of our study is the sample size which is difficult to garner in a single center study when recruitment of subjects occurs in the first trimester and the incidence of subsequently developing APOs is rather low in the general population, namely 9–11% in the case of GDM and 3–5% in the case of PE among pregnant subjects [62–64]. Due to this limitation, we were unable to assign some subjects to testing and others to training groups, making the prediction modelling a proof-of-concept. While EFIRM paralleled RNA-seq in detecting multiple differentially abundant transcripts, detection of corresponding proteins proved more challenging. While optimization of EFIRM to overcome differences due to ranges in affinities of commercially available protein detecting antibodies may be explored, other biological factors beyond technological improvements may have a greater influence, playing a major contributory role. These factors consist of protein sizes dictating the amounts filtered, secreted and re-absorbed by kidneys, and the more commonly encountered true discordance, due to differences between transcription rates and various post-transcriptional processes in the case of certain classes of genes [49].

We conclude that a panel of transcripts are differentially abundant in urine of pregnant subjects during early gestation that can be detected rapidly by EFIRM with the potential ability to distinguish GDM, PE and gHTN from control pregnancies. Most importantly, our present novel findings must be validated in a separate cohort of subjects to assign reliability of predictive capabilities prior to deployment in the clinical setting. EFIRM presents future possibilities of combining machine learning/artificial intelligence to enhance model accuracy towards promoting it in much larger populations as a routine screening tool for prenatal care. Such future steps will pave the way for developing interventions in sub-populations representing endophenotypes, thereby changing management strategies for these high-risk pregnancies and altering the course of adverse pregnancy outcomes.

## Supplementary Material

Supplementary material

**Supplementary Information** The online version contains supplementary material available at https://doi.org/10.1007/s43032-026-02133-4.

## Figures and Tables

**Table 1:** Probe design for detection of transcripts in urine samples

	EFIRM Capture Probe (5’– 3’)	EFIRM Detector probe (5’– 3’)

HLADPB1	AAAAAAAGAAATAAACAAATAA AACAATAACAAATAAAAAAAAA CAAATAAACAATAAAAAAAAAC AATTGTCCTGGAGCCAGATGC	TAACGAA ACACAGC AAATGCT TTTCCTAA G-biotin
GSDMD	AAAAAAAGAAATAAACAAATAA AACAATAACAAATAAAAAAAAA CAAATAAACAATAAAAAAAAAC AATCATGGTCAGTGCCCC	CAGGTAG ACAACAG GGATAGCG AGTTCCG G-biotin
GAPDH	AAAAAAAAAAGAAATAAACAA ATAAAACAATAACAAATAAAAA AAAACAAATAAACAATAAAAAA AAACAATTGATTTTGGAGGGAT	CTCGCTCC TGGAAGAT GGTGATGG GATT-biotin
QSOX1	AAAAAAAGAAATAAACAAATAA AACAATAACAAATAAAAAAAAA CAAATAAACAATAAAAAAAAAC AATTTCTCGCAAAGAATCCATCA	ATCTCCTC CAGCTTGG CAGGCTCC AGTGGGG GA-biotin
MAPK7	AAAAAAAGAAATAAACAAATA AAACAATAACAAATAAAAAAA AACAAATAAACAATAAAAAAA AACAACTGAGAGAGAGGCTGA ATCTG	CCTGGCCA TCCTGTGG CCCATCAG CC-biotin
TERF2	AAAAAAAGAAATAAACAAATA AAACAATAACAAATAAAAAAA AACAAATAAACAATAAAAAAA AACAAAGCTCAGCCTCCATATC AAAG	GAACAGT CTAAATTT TCCCCTT CTTCAAT C-biotin
IL1A	AAAAAAAGAAATAAACAAATAA AACAATAACAAATAAAAAAAAA CAAATAAACAATAAAAAAAAAC AATGTGATGGTTTTGGGTATCTC	AGGCATC TCCTTCAG CAGCACT GGTTGGT C-biotin

The underlined sequences represent the target region, while the non-underlined sequences represent spacers for the EFIRM assay

**Table 2:** Clinical characteristics of the recruited subjects

	Control	GDM	PE	gHTN

Number of samples	15	12	12	11
Ethnicity	Hispanic = 20%; White = 60% Asian = 20%	Hispanic = 25%; While = 16% Asian = 33%, other = 26%	Hispanic = 42%; White = 25% Asian = 17% Other = 16%	Hispanic = 9% White = 55% Asian = 9% Other = 27%
Glucose (mg/dl)	95.06 ± 4.65	158.4 ± 14.26* (p = 0.00001)	110.83 ± 5.58	114.36 ± 8.49
Glucose (A1C)	5.45 ± 0.05	5.59 ± 0.20	5.76 ± 0.25	5.31 ± 0.11
Pregnancy BMI	25.80 ± 5.34	24.53 ± 1.24	27.45 ± 1.81	26.67 ± 2.53
BMI classification	NORM = 58.3%; OBS = 25%; OVWT = 16.6%	NORM = 27%; OBS = 64%; OVWT = 9%	NORM = 41.6%; OBS = 33.3%; OVWT = 25%	NORM = 60%; OBS = 10%; OVWT = 30%
Blood pressure during 1 st trimester (~ 12–15 weeks gestation)				
Blood pressure systolic (mm Hg)	116 ± 2.96	117 ± 2.74	121 ± 3.95	118.45 ± 2.69
Blood pressure diastolic (mm Hg)	73.6 ± 1.68	76.2 ± 1.38	75.3 ± 4.06	76.81 ± 1.93
Blood pressure mean	87.8 ± 2.03	89.9 ± 1.62	90.5 ± 3.80	90.69 ± 2.08
Blood pressure during 2nd/3rd trimester				
Blood pressure systolic (mm Hg)	117 ± 2.25	104 ± 9.72	148 ± 4.42* (*p* = 0.0007)	145.63 ± 3.0* (*p* < 0.001)
Blood pressure diastolic (mm Hg)	70.2 ± 5.34	65.3 ± 6.10	90.3 ± 3.69* (p = 0.023)	94.27 ± 3.44* (*p* < 0.01)
Blood pressure mean	83.3 ± 6.24	78.1 ± 7.24	110 ± 2.97* (*p* = 0.004)	111.39 ± 2.33* (*p* < 0.01)
Birth Wt. classification	SGA = 0% AGA = 87% LGA = 13%	SGA = 9% AGA = 73% LGA = 18%	SGA = 17% AGA = 83% LGA = 0%	SGA = 9% AGA = 82% LGA = 9%
Birth Wt. (gm)	3437.93 ± 110.83	3517.90 ± 163.20	3285 ± 129.40	3046 ± 92.47
Gestational age at delivery	39.50 ± 0.36 (37W – 41 W)	38.88 ± 0.21 (37W1d – 39 W 4d)	38.94 ± 0.42 (37W1d – 40 W 6d)	37.94 ± 0.24 (37W – 41 W 2d)
24 h protein (mg)	26.75 ± 14.3	13.7 ± 1.7	120.41 ± 61.7	11.7 ± 3.19
Protein/Creatinine	0.23 ± 0.03	0.2 ± 0.1	1.88 ± 0.77 (*p* < 0.05)	0.54 ± 0.11
AST	20.25 ± 2.32	15 ± 0.89	29.2 ± 5.53	21 ± 1.88
ALT	20.5 ± 2.88	13.4 ± 1.33	26.1 ± 8.54	17.63 ± 2.59
Baby sex (male-to-female ratio)	0.875	1.5	1.4	2.6
Platelets	208.86 ± 15.55	221.08 ± 13.27	216.91 ± 17.94	209.54 ± 16.05

*p*-values are shown in comparison to the normal group

*BMI* Body mass index, *NORM* normal, *OBS* obesity, *OVWT* overweight, *AGA* appropriate for gestational age, *SGA* small for gestational age, *LGA* large for gestational age, *W* gestational weeks, *AST* aspartate aminotransferase, *ALT* alanine aminotransferase

**Table 3:** Values obtained from mRNA abundance: Area under the curves (AUCs) for adverse pregnancy outcomes (APOs), sensitivity, specificity, positive predictive values (PPV), and negative predictive values (NPV) obtained from urine mRNAs abundance

APOs	AUC (*p*-value)	Sensitivity	Specificity	PPV	NPV

GDM	0.95 (< 0.06)	0.95	0.62	0.68	0.95
PE	0.92 (0.11)	0.91	0.62	0.66	0.90

## Data Availability

All raw sequence data has been uploaded into the publicly available database available at GEO. All RNA-seq and EFIRM analyses are included as Excel files within a BOX folder named “Raw Data” at https://uclahs.box.com/s/v3qt7tjdggydot5k9vqaw2x3vb9h8rhl

## References

[1] Brislane A, Davenport MH, Steinback CD. The sympathetic nervous system in healthy and hypertensive pregnancies: physiology or pathology? Exp Physiol. 2023;108(10):1238–44. 10.1113/EP089665. 36459575 PMC10988427

[2] Umer A, Kanwal S, Ahmad A, Fatima A, Fatima A, Farooq F, Maternal and neonatal outcomes associated with preeclampsia and gestational diabetes mellitus: a retrospective cohort study. Cureus. 2025;17(6):e86120. 10.7759/cureus.86120. 40672040 PMC12265387

[3] Granger JP, Alexander BT, Bennett WA, Khalil RA. Pathophysiology of pregnancy-induced hypertension. Am J Hypertens. 2001;14(6 Pt 2):178S–S185. 10.1016/s0895-7061(01)02086-6. 11411754

[4] Wagner SJ, Barac S, Garovic VD. Hypertensive pregnancy disorders: current concepts. J Clin Hypertens (Greenwich). 2007;9(7):560–6. 10.1111/j.1524-6175.2007.06695.x. 17617769 PMC8109890

[5] Jarvis SS, Shibata S, Bivens TB, Okada Y, Casey BM, Levine BD, Sympathetic activation during early pregnancy in humans. J Physiol. 2012;590(15):3535–43. 10.1113/jphysiol.2012.228262. 22687610 PMC3547268

[6] Karkia R, Giacchino T, Shah S, Gough A, Ramadan G, Akolekar R. Gestational diabetes mellitus: association with maternal and neonatal complications. Medicina (Kaunas). 2023. 10.3390/medicina59122096. PMC10744613 38138200

[7] Shalev-Rosenthal Y, Hadar E, Rosenthal A, Ram S, Shalev-Ram H, Danieli-Gruber S, Once gestational diabetes, always gestational diabetes? Maternal and neonatal outcomes of pregnancies with gestational diabetes preceding non gestational diabetes pregnancy- a retrospective cohort study. Am J Obstet Gynecol. 2025. 10.1016/j.ajog.2025.07.010. 40645473

[8] Lachmann EH, Fox RA, Dennison RA, Usher-Smith JA, Meek CL, Aiken CE. Barriers to completing oral glucose tolerance testing in women at risk of gestational diabetes. Diabet Med. 2020;37(9):1482–9. 10.1111/dme.14292. 32144795 PMC8641378

[9] Farrar D, Duley L, Dowswell T, Lawlor DA. Different strategies for diagnosing gestational diabetes to improve maternal and infant health. Cochrane Database Syst Rev. 2017;8(8):CD007122. 10.1002/14651858.CD007122.pub4. 28832911 PMC6483546

[10] Helton MR, Arndt J, Kebede M, King M. Do low-risk prenatal patients really need a screening glucose challenge test? J Fam Pract. 1997;44(6):556–61. 9191628

[11] Ives CW, Sinkey R, Rajapreyar I, Tita ATN, Oparil S. Preeclampsia-pathophysiology and clinical presentations: JACC state-of-the-art review. J Am Coll Cardiol. 2020;76(14):1690–702. 10.1016/j.jacc.2020.08.014. 33004135

[12] Duley L The global impact of pre-eclampsia and eclampsia. Semin Perinatol. 2009;33(3):130–7. 10.1053/j.semperi.2009.02.010. 19464502

[13] Dathan-Stumpf A, Rieger A, Verlohren S, Wolf C, Stepan H. sFlt-1/PlGF ratio for prediction of preeclampsia in clinical routine: a pragmatic real-world analysis of healthcare resource utilisation. PLoS One. 2022;17(2):e0263443. 10.1371/journal.pone.0263443. 35202416 PMC8870556

[14] Chang KJ, Seow KM, Chen KH. Preeclampsia: recent advances in predicting, preventing, and managing the maternal and fetal life-threatening condition. Int J Environ Res Public Health. 2023. 10.3390/ijerph20042994. PMC9962022 36833689

[15] Baetens M, Van Gaever B, Deblaere S, De Koker A, Meuris L, Callewaert N, Advancing diagnosis and early risk assessment of preeclampsia through noninvasive cell-free DNA methylation profiling. Clin Epigenetics. 2024;16(1):182. 10.1186/s13148-024-01798-5. 39695764 PMC11656954

[16] Rasmussen M, Reddy M, Nolan R, Camunas-Soler J, Khodursky A, Scheller NM, RNA profiles reveal signatures of future health and disease in pregnancy. Nature. 7893;601(7893):422–7. 10.1038/s41586-021-04249-w. PMC8770117 34987224

[17] Del Vecchio G, Li Q, Li W, Thamotharan S, Tosevska A, Morselli M, Cell-free DNA methylation and transcriptomic signature prediction of pregnancies with adverse outcomes. Epigenetics. 2021;16(6):642–61. 10.1080/15592294.2020.1816774. 33045922 PMC8143248

[18] Ghosh S, Thamotharan S, Fong J, Lei MYY, Janzen C, Devaskar SU. Circulating extracellular vesicular microRNA signatures in early gestation show an association with subsequent clinical features of pre-eclampsia. Sci Rep. 2024;14(1):16770. 10.1038/s41598-024-64057-w. 39039088 PMC11263608

[19] Thamotharan S, Ghosh S, James-Allan L, Lei MYY, Janzen C, Devaskar SU. Circulating extracellular vesicles exhibit a differential miRNA profile in gestational diabetes mellitus pregnancies. PLoS ONE. 2022;17(5):e0267564. 10.1371/journal.pone.0267564. 35613088 PMC9132306

[20] Tuntivararut P, Raungrongmorakot K, Chaiyasit N, Yuenyongdechawat N, Chaemsaithong P. Urine Congo red test for the detection of preeclampsia in pregnant women presenting with suspected preeclampsia. J Matern Fetal Neonatal Med. 2024;37(1):38538334, 2332787. 10.1080/14767058.2024.2332787. 38538334

[21] Butler SA, Khanlian SA, Cole LA. Detection of early pregnancy forms of human chorionic gonadotropin by home pregnancy test devices. Clin Chem. 2001;47(12):2131–6. 11719477

[22] Gnoth C, Johnson S. Strips of hope: accuracy of home pregnancy tests and new developments. Geburtshilfe Frauenheilkd. 2014;74(7):661–9. 10.1055/s-0034-1368589. 25100881 PMC4119102

[23] Li F, Wei F, Huang WL, Lin CC, Li L, Shen MM, Ultra-short circulating tumor DNA (usctDNA) in plasma and saliva of non-small cell lung cancer (NSCLC) patients. Cancers (Basel). 2020. 10.3390/cancers12082041. PMC7464208 32722209

[24] Qian J, Xia J, Chiang S, Liu JF, Li K, Li F, Rapid and comprehensive detection of viral antibodies and nucleic acids via an acoustofluidic integrated molecular diagnostics chip: AIMDx. Sci Adv. 2025;11(3):eadt5464. 10.1126/sciadv.adt5464. 39813350 PMC11734728

[25] Wei F, Lin CC, Joon A, Feng Z, Troche G, Lira ME, Noninvasive saliva-based EGFR gene mutation detection in patients with lung cancer. Am J Respir Crit Care Med. 2014;190(10):1117–26. 10.1164/rccm.201406-1003OC. 25317990 PMC5447327

[26] Wei F, Strom CM, Cheng J, Lin CC, Hsu CY, Soo Hoo GW, Electric field-induced release and measurement liquid biopsy for noninvasive early lung cancer assessment. J Mol Diagn. 2018;20(6):738–42. 10.1016/j.jmoldx.2018.06.008. 30309763 PMC6198245

[27] Wei F, Yu P, Cheng J, Li F, Chia D, Wong DTW. Single-droplet microsensor for ultra-short circulating EFGR mutation detection in lung cancer based on multiplex EFIRM liquid biopsy. Int J Mol Sci. 2023. 10.3390/ijms241210387. PMC10299723 37373532

[28] ACOG Practice Bulletin No. 190 Summary: gestational diabetes mellitus. Obstet Gynecol. 2018;131(2):406–8. 10.1097/AOG.0000000000002498. 29370044

[29] McLaren RA, Atallah F, Persad VVD, Narayanamoorthy S, Gougol N, Silver M, Pregnancy outcomes among women with American College of Cardiology- American Heart Association defined hypertension. J Matern Fetal Neonatal Med. 2021;34(24):4097–102. 10.1080/14767058.2019.1704250. 31875736

[30] ACOG Committee on Obstetric Practice. ACOG practice bulletin. Diagnosis and management of preeclampsia and eclampsia. Number 33, January 2002. American College of Obstetricians and Gynecologists. Int J Gynaecol Obstet. 2002;77(1):67–75. 12094777

[31] ACOG practice bulletin. Diagnosis and management of preeclampsia and eclampsia. Obstet Gynecol. 2002;99(1):159–67. 10.1016/s0029-7844(01)01747-1. 16175681

[32] Plaisier SB, Taschereau R, Wong JA, Graeber TG. Rank-rank hypergeometric overlap: identification of statistically significant overlap between gene-expression signatures. Nucleic Acids Res. 2010;38(17):e169. 10.1093/nar/gkq636. 20660011 PMC2943622

[33] Friedman J, Hastie T, Tibshirani R. Regularization paths for generalized linear models via coordinate descent. J Stat Softw. 2010;33(1):1–22. 20808728 PMC2929880

[34] Sachs MC. PlotROC: a tool for plotting ROC curves. J Stat Softw. 2017. 10.18637/jss.v079.c02. PMC6347406 30686944

[35] Li F, Wei F, Grogan TR, Elashoff DE, Vu D, Vigerust DJ, Proficiency testing of epidermal growth factor receptor mutations detection in saliva using Spectrum Saliva Collector (SDNA-1000) and preservative solution detected by electric field-induced release and measurement. Biopreserv Biobank. 2022;20(5):461–4. 10.1089/bio.2022.0093. 35878053 PMC9603249

[36] Benjamini Y, Hochberg Y. Controlling the false discovery rate: a practical and powerful approach to multiple testing. J Roy Stat Soc: Ser B (Methodol). 1995;57(1):289–300.

[37] Du R, Bai Y, Li L, Shao Y, Wu N. Insulin resistance-induced mitochondrial dysfunction and pyroptosis in trophoblasts: protective role of metformin. BMC Pregnancy Childbirth. 2025;25(1):293. 10.1186/s12884-025-07419-0. 40089682 PMC11910837

[38] Liu Z, Chen Y, Mei Y, Yan M, Liang H. Gasdermin D-mediated pyroptosis in diabetic cardiomyopathy: molecular mechanisms and pharmacological implications. Molecules. 2023. 10.3390/molecules28237813. PMC10708146 38067543

[39] Wong GP, Hartmann S, Simmons DG, Ellis S, Nonn O, Cannon P, Trophoblast side-population markers are dysregulated in preeclampsia and fetal growth restriction. Stem Cell Rev Rep. 2024;20(7):1954–70. 10.1007/s12015-024-10764-w. 39028417 PMC11445292

[40] Ilani T, Reznik N, Yeshaya N, Feldman T, Vilela P, Lansky Z, The disulfide catalyst QSOX1 maintains the colon mucosal barrier by regulating Golgi glycosyltransferases. EMBO J. 2023;42(2):e111869. 10.15252/embj.2022111869. 36245281 PMC9841341

[41] Rudolf J, Pringle MA, Bulleid NJ. Proteolytic processing of QSOX1A ensures efficient secretion of a potent disulfide catalyst. Biochem J. 2013;454(2):181–90. 10.1042/BJ20130360. 23713614 PMC3749868

[42] Wang Z, Wu X. Abnormal function of telomere protein TRF2 induces cell mutation and the effects of environmental tumor-promoting factors (Review). Oncol Rep. 2021;46(2). 10.3892/or.2021.8135. PMC8273685 34278498

[43] Zhang S, Hemmerich P, Grosse F. Nucleolar localization of the human telomeric repeat binding factor 2 (TRF2). J Cell Sci. 2004;117(Pt 17):3935–45. Epub 20040720. 10.1242/jcs.01249. 15265990

[44] Hua L, Chen W, Meng Y, Qin M, Yan Z, Yang R, The combination of DNA methylome and transcriptome revealed the intergenerational inheritance on the influence of advanced maternal age. Clin Transl Med. 2022;12(9):e990. 10.1002/ctm2.990. 36103411 PMC9473489

[45] Plooster M, Rossi G, Farrell MS, McAfee JC, Bell JL, Ye M, Schizophrenia-linked protein tSNARE1 regulates endosomal trafficking in cortical neurons. J Neurosci. 2021;41(45):9466–81. 10.1523/JNEUROSCI.0556-21.2021. 34642214 PMC8580139

[46] Dinarello CA. Overview of the IL-1 family in innate inflammation and acquired immunity. Immunol Rev. 2018;281(1):8–27. 10.1111/imr.12621. 29247995 PMC5756628

[47] Rabolli V, Badissi AA, Devosse R, Uwambayinema F, Yakoub Y, Palmai-Pallag M, The alarmin IL-1α is a master cytokine in acute lung inflammation induced by silica micro- and nanoparticles. Part Fibre Toxicol. 2014;11:69. 10.1186/s12989-014-0069-x. 25497724 PMC4279463

[48] Bokenkamp A Proteinuria-take a closer look! Pediatr Nephrol. 2020;35(4):533–41. Epub 20200110. 10.1007/s00467-019-04454-w. 31925536 PMC7056687

[49] Prabahar A, Zamora R, Barclay D, Yin J, Ramamoorthy M, Bagheri A, Johnson SA, Badylak S, Vodovotz Y and Jiang P (2024). “Unraveling the complex relationship between mRNA and protein abundances: a machine learning-based approach for imputing protein levels from RNA-seq data.” NAR Genom Bioinform 6(1): lqae019. 38344273 10.1093/nargab/lqae019 PMC10858678

[50] Agarwal NR, Kachhawa G, Oyeyemi BF, Bhavesh NS. Urine metabolomics reveals overlapping metabolic associations between preeclampsia and gestational diabetes. Indian J Clin Biochem. 2024;39(3):356–64. Epub 20221206. 10.1007/s12291-022-01103-2. 39005861 PMC11239642

[51] Aleidi SM, Al Fahmawi H, AlMalki RH, Al Mogren M, Alwahsh M, Mujammami M, Untargeted metabolomics profiling of gestational diabetes mellitus: insights into early diagnosis and metabolic pathway alterations. Front Mol Biosci. 2024;11:1485587, 1485587. 10.3389/fmolb.2024.1485587. 39764206 PMC11700826

[52] Chen S, Li J, Ren S, Gao Y, Zhou Y, Xuan R. Expression and clinical significance of short-chain fatty acids in pregnancy complications. Front Cell Infect Microbiol. 2022;12:1071029. Epub 20230112. 10.3389/fcimb.2022.1071029. 36710961 PMC9876977

[53] Kim JE, Kim SY, Cheong JC, Kim JY. A dilute-and-shoot LC-MS/MS determination of low-dosage third-generation antipsychotics and their metabolites in urine using an ultra-short column. J Chromatogr B Analyt Technol Biomed Life Sci. 2025;1255:124523. Epub 20250212. 10.1016/j.jchromb.2025.124523. 39955960

[54] Lei R, Huo R, Mohan C. Current and emerging trends in point-of-care urinalysis tests. Expert Rev Mol Diagn. 2020;20(1):69–84. Epub 20191212. 10.1080/14737159.2020.1699063. 31795785 PMC7365142

[55] Yeasmin SAG, Onder A, Yan E, Yildiz UH, Palaniappan A, Liedberg B. Current trends and challenges in point-of-care urinalysis of biomarkers in trace amounts. TrAC Trends Anal Chem. 2022. 10.1016/j.trac.2022.116786.

[56] Sequeira-Antunes B, Ferreira HA. Urinary biomarkers and point-of-care urinalysis devices for early diagnosis and management of disease: a review. Biomedicines. 2023. 10.3390/biomedicines11041051. PMC10135468 37189669

[57] Thomas CE, Sexton W, Benson K, Sutphen R, Koomen J. Urine collection and processing for protein biomarker discovery and quantification. Cancer Epidemiol Biomarkers Prev. 2010;19(4):953–9. 10.1158/1055-9965.EPI-10-0069. 20332277 PMC2852495

[58] Ng KW, Chaturvedi N, Cote GL, Fisher SA, Mabbott S. Biomarkers and point of care screening approaches for the management of preeclampsia. Commun Med. 2024;4(1):208. 10.1038/s43856-024-00642-4. 39433973 PMC11493996

[59] Liu X, Xu J, Liu C, Li X. CercaTest Red, a novel urine-based point-of-care test for the detection of preeclampsia. Curr Med Res Opin. 2024;40(3):395–401. 10.1080/03007995.2024.2314245. 38321953

[60] Verlohren S, Galindo A, Schlembach D, Zeisler H, Herraiz I, Moertl MG, An automated method for the determination of the sFlt-1/PIGF ratio in the assessment of preeclampsia. Am J Obstet Gynecol. 2010;202(2):e1–11. 10.1016/j.ajog.2009.09.016. 19850276

[61] Thadhani R, Lemoine E, Rana S, Costantine MM, Calsavara VF, Boggess K, Circulating angiogenic factor levels in hypertensive disorders of pregnancy. NEJM Evid. 2022;1(12):EVI–Doa2200161. 10.1056/EVIDoa2200161. 38319832

[62] Ayyash MK, McLaren RJ Jr, Shaman M, Al-Kouatly HB. Trends in preeclampsia risk factors in the US from 2010 to 2021. JAMA. 2010;332(2):167–9. 10.1001/jama.2024.8931. PMC11165405 38857021

[63] Eades CE, Burrows KA, Andreeva R, Stansfield DR, Evans JM. Prevalence of gestational diabetes in the United States and Canada: a systematic review and meta-analysis. BMC Pregnancy Childbirth. 2024;24(1):204. 10.1186/s12884-024-06378-2. 38491497 PMC10941381

[64] Wheeler SM, Myers SO, Swamy GK, Myers ER. Estimated prevalence of risk factors for preeclampsia among individuals giving birth in the US in 2019. JAMA Netw Open. 2019;5(1):e2142343. 10.1001/jamanetworkopen.2021.42343. PMC8728614 34982156

