# Supplementary material for: Electric Field-Induced Release and Measurement Liquid Biopsy of Urinary Transcriptomics and Key Proteins from Normal and High-risk Pregnant Women
Source: Reprod Sci. 2026 Jun 19;33(7):1379–93. doi: 10.1007/s43032-026-02133-4 (PMC13415046; doi:10.1007/s43032-026-02133-4)
Supplement: Supplementary file 1 — Supplementary file1 (DOCX 3389 KB) [file 43032_2026_2133_MOESM1_ESM.docx]

**Supplemental Figures**

**Figure 1: Heat maps** demonstrating the differentially abundant transcripts identified by RNA-seq between GDM (**A**), PE (**B**), or gHTN (**C**) versus CON pregnant subjects (color key: blue=decreased, red=increased), obtained from urine collected during first (T1) and second (T2) trimesters. Transcripts are differentially abundant based on log2FC >1 and padj < 0.05.


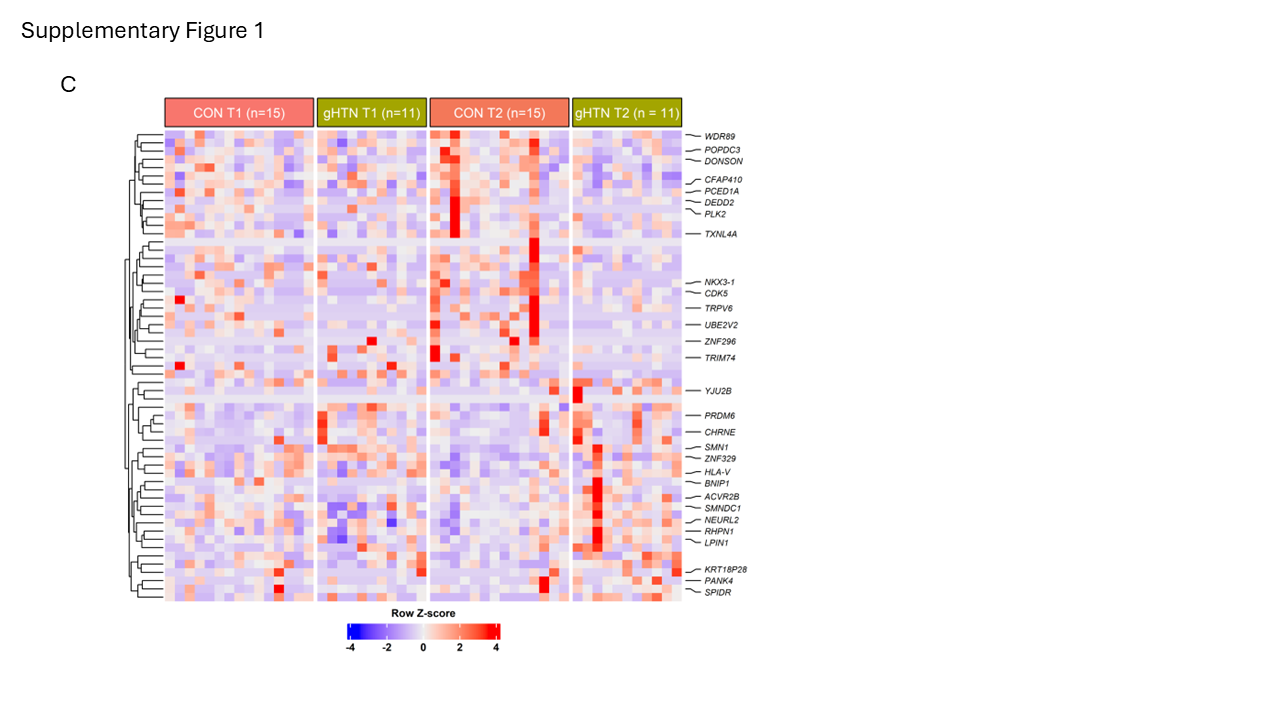

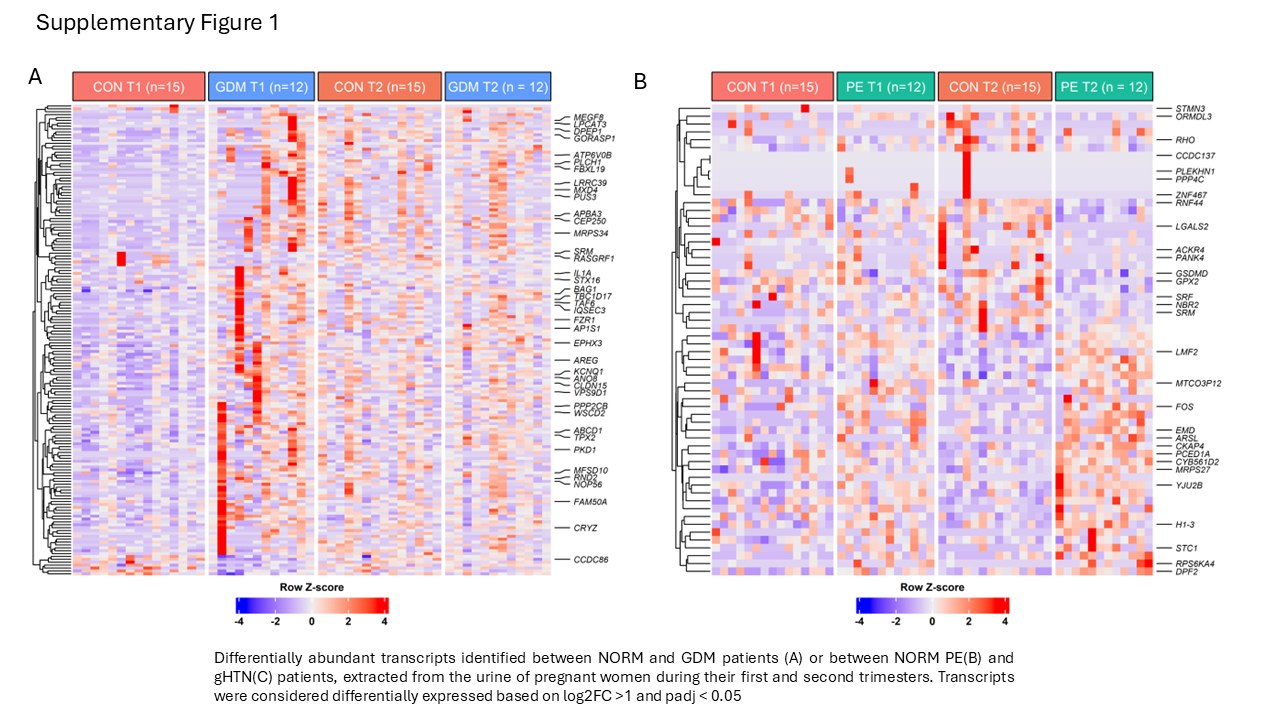


**
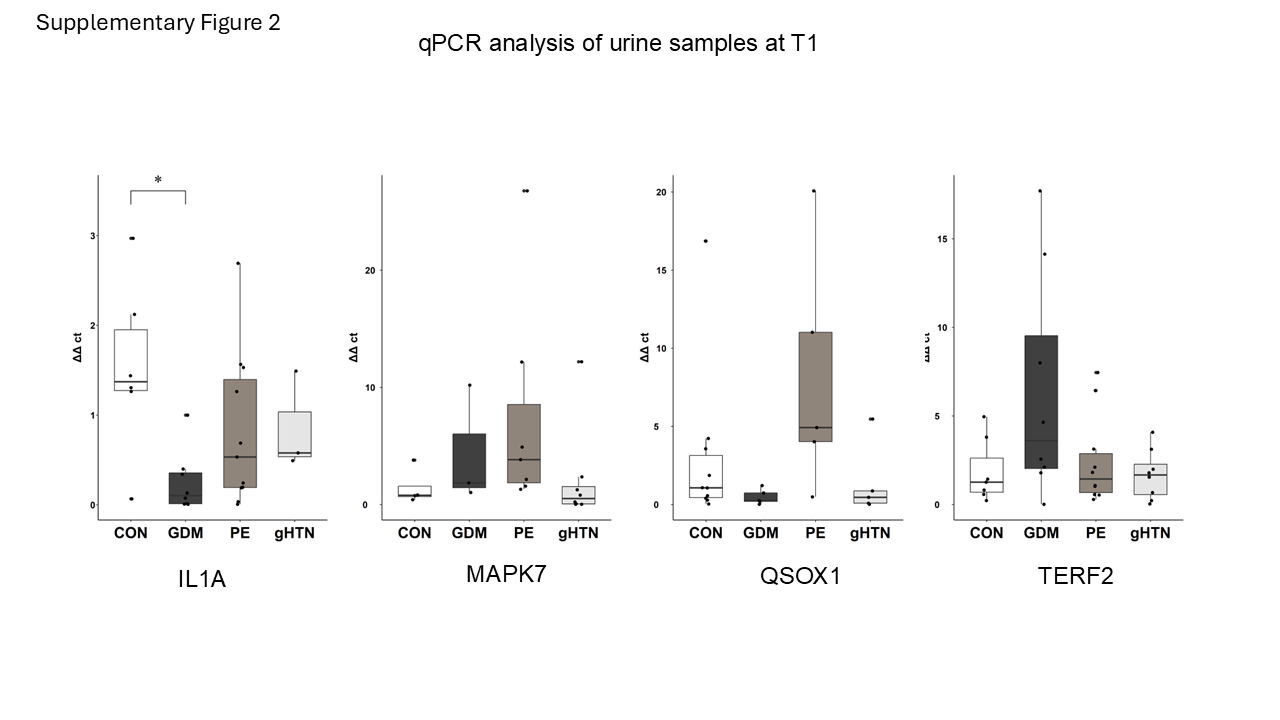
Figure 2: Reverse-transcription-qPCR measurements** shown as box and whiskers plots of transcripts IL1a, MAPK7, QSOX1 and TERF2 obtained from urine collected during the first trimester (T1) from CON, GDM, PE, and gHTN pregnant subjects. *p<0.05 versus CON.

**
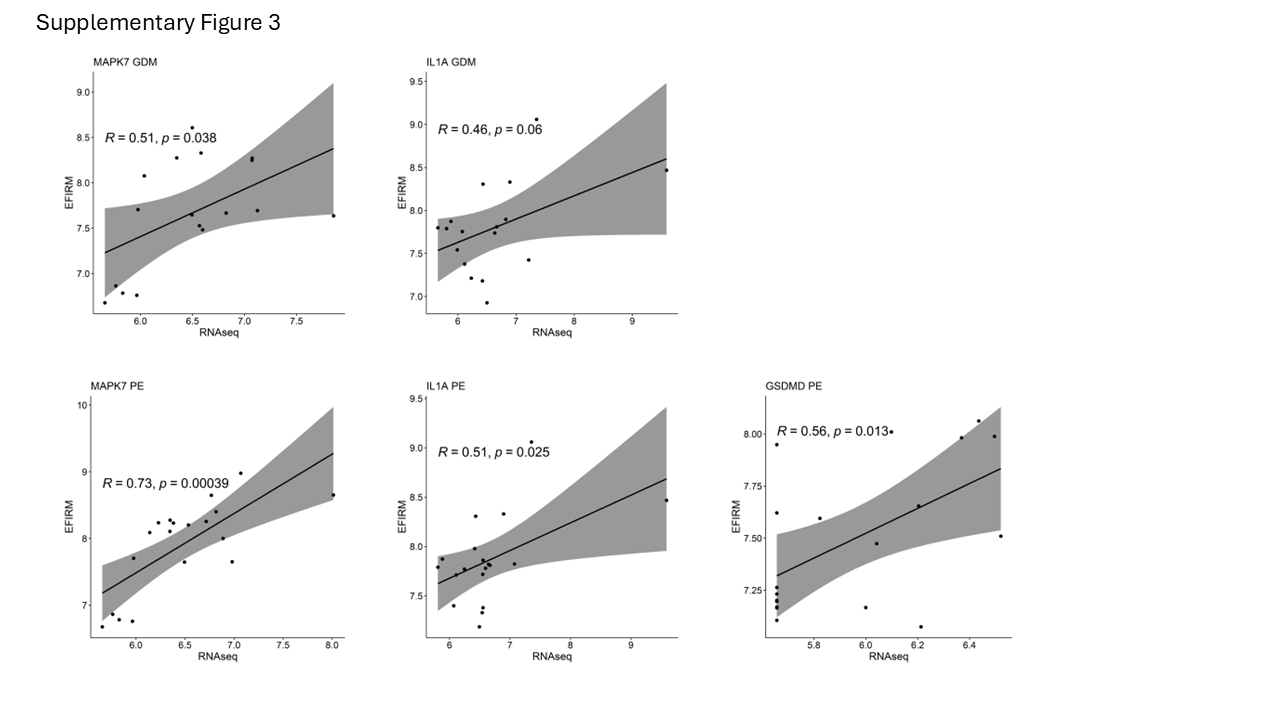
Figure 3: Correlation coefficients** (R) and p-values are shown between RNA-seq and EFIRM methods in detection of urinary IL1a, MAPK7 transcripts in GDM and IL1A, MAPK7 and GSDMD in PE during T1 of pregnancy.
